# Pruritus Ani1Read before the Buffalo Academy of Medicine, Section on Surgery, April 28, 1902.

**Published:** 1902-06

**Authors:** George L. Brown

**Affiliations:** Buffalo, N. Y.


### Pruritus Ani.[1]

By GEORGE L. BROWN, M. D.. Buffalo, N. Y.

THIS condition presents more difficulties of diagnosis, more complications and combination of symptoms, than almost any of the diseases of the anal region. It is one of the most obstinate, annoying, vexatious and loathsome complaints known to suffering humanity; it affects both sexes, and all classes and conditions alike, the rich and the poor, the illy nourished and the overfed, the sedentary professional man, and the toiling day-laborer, the woman of society, and the woman who earns her livelihood over the washtub. No age or position is exempt from its infliction, or secure from its tortures. It is not an idiopathic disease, but always a symptomatic affection. In many cases its pathology and causation are very obscure. Ordinary, well-defined cases are recognised without difficulty, there being certain symptoms common to each; it is the obscure and complicated conditions that occasion mistakes of diagnosis.

The earlier manifestations are usually of a trivial character. The first noticeable indication of its presence is an itching sensation, on or about the verge of the anus, which is found to be more pronounced at night, especially after the patient becomes warm in bed. The itching comes on so gradually and insidiously, that the sufferer gives but little attention to it, but in time becomes so vexatious and unbearable that the patient contracts the habit of scratching the affected parts, in vain effort to obtain relief, which habit grows more confirmed and annoying the further the case progresses, and is frequently practised unconsciously during sleep. This intolerable itching, which is the characteristic feature of pruritus ani and is the burden of the patient's complaints, is one of the most intractable of the rectal troubles, especially so when the cause is not apparent, or readily discovered, as is many times the case. The actual pain experienced is not severe, but the itching is less easily endured, and, together with inability to secure refreshing and restful sleep for nights in succession, the nerves and moral stamina of the sufferer are worn down, so that in frequent cases life becomes such a burden that the victim becomes moody, melancholy and despondent, and contemplates suicide as the only sure and certain relief. The itching gradually spreads about the surrounding parts of the anal verge, extending up

1. Read before the Buffalo Academy of Medicine, Section on Surgery, April 28, 1902.

the rectum, over the integument of the buttocks, scrotum, vulva, perineum, and to more remote parts of the body, such as the nose, ears and eyelids, and in fact wherever the sufferer has carried the germ or exudation with the hands or fingernails.

This affection is irregularly paroxysmal, and in the intervals there is no itching or other disturbance. In advanced cases certain morbid changes occur around the rectal orifice, in consequence of the patient's strenuous efforts to relieve the intolerable itching, which, while giving momentary relief, excoriates the naturally thin skin, exaggerates the secretions of the parts, and if the irritation continues, fissures and even ulcerations are formed. Vesicles appear which discharge an irritating sanies, that sometimes removes the cuticle and leaves the parts raw and bleeding, at other times smooth, glassy and shining, frequently bleaching the skin white.

The causes of this distressing ailment are many and varied, among which are proctitis, the overstretching of the anal orifice in the forcible discharge of costive stools, although the itching may not manifest itself for some time; pocket piles—papulæ—polypi on the walls of the rectum and bowel, ulcers and fissures, irritable pile tumors, all forms of hemorrhoids, inverted or ingrowing skin or hairs in the rectum; all kinds of ascarides, eczema, erythema, herpes, pityriasis, acnæ, lichen, prurigo, urticaria, verucca, tinea, pediculosis pubis, sexual irregularities, tuberculous conditions, venereal diseases, the various uterine and ovarian troubles to which woman is heir; also some lesions of the sensory fibers surrounding the verge of the anus, and disease of the spinal cord or brain.

Among causes not generally recognised as such are rectal inflammations, intestinal autointoxication, masturbational neuroses, dry scybalæ adhering to the lining of the bowel, intemperate use of malt liquors, irritating secretions, caused by improper food, or by some diseased organ of nutrition, which does not perform its function in proper manner. Other causes are sitting upon cold or damp ground, wood, stone or metal, the use of leather covered or upholstered seats, and improper materials for toilet purposes, especially paper containing printer's ink, the habit of sitting upon a closet for a longer time than is absolutely necessary for the purposes of defecation, the use of highly spiced foods, imperfect mastication, the insufficient supply of water for the wants of the system and eating at irregular intervals.

Proctitis—by far the most prolific cause of pruritus ani—is produced by use of drastic cathartics, septic infection, or to a specific poison, such as dysentery, gonorrhea, syphilis, tuberculosis or cancer. It is brought on by the use of improper articles of diet, neglect of proper attention to the wants of nature, thereby inciting constipation, obstipation and impacted feces, which result in inflammation and ulceration extending into the surrounding tissue, and this tissue becoming infiltrated, breaks down into abscesses containing fetid pus, which communicates with burrowing sinuses and fistulous tracts, leading into the surrounding parts, forming blind pouches and sacciform enlargement about the rectum.

Periproctitis may exist without any antecedent rectal ulceration, especially in connection with the above diseases. This inflammation comes on so insidiously and gradually, as frequently to assume a chronic form, without the person afflicted having any knowledge of its presence, until the severe pruritus ani becomes so troublesome and continuous, that the services of a physician are deemed necessary and the true cause determined.

When an examination exposes blind internal fistulous tracts, ending in rugæ and indurated tabs about the anus, we find that upon removal of the investing cuticle, cavities containing polypi, and prong-shaped growths, with fork or thorn-like points,—conditions that are accountable for a great deal of the itching,—the itching ceases.

Rectitis, the result of costive stools and the consequent strain upon the muscles in their forcible evacuation, is accountable for many of the troubles of this nature, among which are what are known as pocket-piles. They are the sacs discovered by Dr. Horner and have been named sacculi Horneri. These sacs, pouches or pockets, catch small particles of fecal matter and foreign substances, such as seeds, cherry pits, nail heads, spiculæ of bone, toothpicks, etc., which cause congestion and frequently ulceration. They appear to discharge at irregular intervals a glairy fluid and while discharging, the itching, burning and gnawing become more pronounced and aggravated. This discharge in some cases is of a very offensive character, even when the most scrupulous cleanliness is observed. Gonorrhea is one great cause, and is the origin of the most annoying and perplexing complications of pruritus ani with which the physician has to contend. It may follow the actual spread of the gonorrheal virus into the rectum, producing rectitis, or from the sympathetic irritation of the rectum, perineum or urethra, and, as a result, we find pruritus ani.

Pruritus ani is in some instances an attendant symptom of Bright's disease, diabetes, cancer, gout, jaundice and diseases of the liver. We recall an aggravated case where the sufferer from the intolerable itching was on the verge of insanity. Examination disclosed the presence of a tape-worm, and with its expulsion, the itching and all the unpleasant accompanying symptoms disappeared.

So much obscurity exists in regard to the initial symptoms, and so much variation in the behavior of cases, that our diagnoses must be largely conjectural, therefore no well defined course of treatment can be laid down.

The first object is to at once alleviate the distress of the unfortunate sufferer; and much can be done toward that end. Unfortunately, we possess no specific for this complaint and the treatment must be symptomatic. Many cases prove so obstinate as to be almost or quite incurable. The utmost patience is required of the sufferer to second the intelligent direction of the physician, who will find necessary the most careful study and attention to ascertain and remove the cause.

The long-continued want of refreshing sleep and the depressing effects upon the mind of the patient, arising from the incessant itching, pricking, burning and needle-piercing sensations (as they are variously described by different sufferers) have a tendency to and do impair the general health, lower the capacity for repair of the vital powers, and dull and debase the moral sense.

Among the evil consequences that sometimes arise and at which we can merely point, is the habit of masturbation, as the constant scratching and rubbing the parts produce an orgasm. Other sequelæ will suggest themselves to the experienced practitioner. Many patients will change from one physician to another, and failing to realise as prompt relief as they think they should, change again to become again discouraged, without giving any one physician time and opportunity to effect a cure. Such cases are many and difficult of treatment. They have tried this doctor and that doctor, who gave this and that remedy, which did them no good, and they know that this and that is not applicable to their case. Through advice of well-meaning but ignorant friends they have tried nearly all the remedies known to the pharmacopeia and many not known, from herbs and roots to christian science, and found all of no avail. These patients are not desirable, but such cases call for study and research and invite the best efforts of the intelligent and conscientious physician. The confidence of the patient must be secured and respected; the cause of the trouble must be discovered as early as possible and efforts toward its removal at once instituted.

Palliative remedies must be employed as occasion requires, the general health of the patient improved by administration of proper tonics, the patient must be brought to understand that a cure can only be effected by great exercise of patience on his own part, and faithful adherence to the instructions of his medical adviser. Excesses of all kinds must be avoided, the necessity of cleanliness, of moderate exercise in the open air, and employment to occupy the mind must be insisted upon. Habits and foods likely to produce disturbance or injury to the lower part of the intestinal canal must be avoided.

The infinite variety of complications existing with pruritus ani, and the multitude of palliative and remedial agents at our command in dealing with its combinations, prevent my giving in detail the treatment of all the affections which give rise to its presence, but I will confine myself to some of the procedures employed as the result of my own experience.

The first and greatest agent in all cases is cleanliness. Probably no one measure is productive of as prompt though temporary relief, as the application to the itching surfaces of water as hot as can be borne. A wide choice of palliatives is within our reach for such purposes, among which are Fuller's earth, sulphur, starch, zinc oxide, bismuth, chalk, magnesia, camphor, ichthyol, mercury in its various forms, campho-phenique, carbolic acid, salicylic acid, listerine, glycerin, alcohol, hydrastis, Pond's extract, opium preparations, orthoform, cocaine and eucaine, which may be applied as the conditions of the case call for, and the occasion may demand. These articles should be combined with a base of petrolatum, or other bland media, carefully avoiding the use of everything of an oily or greasy nature. In cases of proctitis, arising from whatever cause, we should make frequent applications to the inflamed and congested surfaces of glycerole of tannin, thiol, and astringents, and direct the use by enema of one or two drams of raw linseed oil, or a weak solution of zinci acetatis or boracic acid twice daily, with instructions to retain the same, or the introduction of suppositories composed of hydrastis, belladonna with cocoa butter, or soothing alteratives, together with the internal administration of tonics.

In cases of proctitis with gonorrheal complications, we find balsam copaibæ to be the sovereign remedy, which may be administered by mouth or rectum. Chian or spirits of turpentine, cubebs, ichthyol, saw palmetto, santal oil, sulphoichthyolate of ammonia, are also remedies of great importance. All papulæ, polypi, excrescences, inverted hairs and ingrowing skin, must be removed. Venereal diseases will require constitutional treatment. Tuberculous sores demand supporting measures, with applications of astringent, antiseptic and stimulating lotions. The radical extirpation of hemorrhoids by dissection, ligation, excision, clamp and actual cautery, or Whitehead's operation is not successful in every case. To obtain satisfactory results, cases must be selected and certain conditions avoided and recommended to palliative treatment.

Such patients as decline to submit to the knife or anesthetics, the senile and the debilitated, and those of a hemorrhagic condition, are susceptible of cure by the application of remedies frequently applied to the diseased tissues, and the use of hypodermic injections. But most physicians in active practice have not the time and patience necessary to devote to those sufferers. When the itching arises from a neurotic condition, or from a diseased condition of the brain, spinal cord or their coverings, bromides, hypnotics and tonics may be prescribed, as the exigencies of the case demand and the indications suggest. Cancer in all its forms must be extirpated and should the radical operation for its removal be unavailable or inoperative, the permanent relief of pruritus ani will be hopeless. All ulcers in the bowel should be opened, curetted and cauterised with acid nitrate of mercury, nitric acid, carbolic acid, permanganate of potash or Churchill's tincture of iodine, and carefully cleansed once or twice daily with peroxide of hydrogen and suppositories of hydrastis, iodoform, or a weak solution of zinci acetatis should be injected and retained; if there be much pain some anodyne should be added.

Fistulæ must be obliterated by laying open and dissecting out the channels, or by the injection of escharotics and cleansing daily with antiseptic washes. We frequently meet patients suffering from impacted feces who have taken "physic and saline cathartics" as they say with excellent results for a continued period, but are still enduring the trouble arising from that condition, and find on examination the obstruction still present, but with a groove worn into the masses, by the liquid passages caused by these remedies.

For this condition I would advise an enema composed of ox-gall, mixed with hot water or castor oil, one ounce, spirits of turpentine, one dram, the yolk of an egg, and hot soap suds made of castile soap, one quart. The patient should be directed to retain this as long as possible and repeat the same frequently. We should aid him by breaking or cutting up the mass with proper instruments. After the patient is relieved it will be desirable to tone up the system, keep the bowels loose, and use intestinal antiseptics, or the same conditions will recur. When due to the presence of worms, treatment for their prompt and effectual removal must be employed.

The numerous skin diseases should receive the treatment they respectively require. The various uterine, ovarian and sexual troubles, diabetes, Bright's disease, gout, jaundice and affections of the liver require careful research and treatment. In cases of rigid and contracted sphincter, dilatation is frequently necessary and will give temporary relief, but, unless the exciting cause be removed, the contraction and itching will return. Anal fissure, the inciting cause of many cases of pruritus ani with which we have to contend, is produced by costive stools, or from spiculæ of bone, nails, seeds, or other foreign substances in the fecal matter, which, in their passage have injured the membrane, or by a polypus growth in the rectum. In such cases the sphincter muscles are contracted, and become so rigid that it is impossible to introduce anything into the bowel without causing great pain.

If an anesthetic is inadmissible, the dilatation of the sphincter must be accomplished by the administration of cocaine hypodermatically, the polypus removed, and the fissure treated with a saturated solution of carbolic acid, permanganate of potash, chloride or iodide of zinc, applied with a pointed glass rod or stick. After such cauterisation, the fissure should be painted with collodion, composed of iodoform, balsam of tolu or fir, or benzoin and sulphuric ether. The torturing pain attending a fissure, does not usually manifest itself until about thirty minutes after an evacuation; an anodyne should, therefore, be inserted immediately after the alvine discharge, thereby obviating the intense suffering, and reserving the strength of the patient. Continuance of this treatment after each evacuation, with frequent applications of soothing lotions, will cause the severity and duration of the pain to grow less day by day, until the process of healing has become complete.

In writing upon a subject on which medical literature is so nearly silent, the writer is to a very great extent dependent upon his own observation and experience, and has, perhaps, given more prominence to some aspects of the subject than their importance may seem to merit. After an experience of many years in the treatment of this and kindred complaints, we believe that we have only attained the threshold of possible knowledge in this and other rectal troubles, and consider it to be one of the most promising fields for original research to be found within the domain of medicine.

200-210 D. S. Morgan Building.

